# Patients’ satisfaction and efficacy of modern conventional hearing aids: A comprehensive analysis of the self-reported user experiences in adult people

**DOI:** 10.1016/j.bjorl.2025.101565

**Published:** 2025-02-05

**Authors:** Daniele Portelli, Cosimo Galletti, Sabrina Loteta, Leonard Freni, Francesco Ciodaro, Angela Alibrandi, Giuseppe Alberti

**Affiliations:** a University of Messina, Department of Adult and Development Age Human Pathology “Gaetano Barresi”, Unit of Otorhinolaryngology, Messina, Italy; b University of Catanzaro “Magna Graecia”, Unit of Otorhinolaryngology, Catanzaro, Italy; c University of Messina, Department of Economics, Unit of Statistical and Mathematical Sciences, Messina, Italy

**Keywords:** Hearing aid, Hearing loss, Personal satisfaction

## Abstract

**Objective:**

Hearing loss is a widespread condition that impacts the quality of life, affecting communication, social interactions, and cognitive functions. This study aimed to evaluate the subjective benefit and satisfaction of using hearing aids among patients since the adoption rate of hearing aids remains low.

**Methods:**

The study retrospectively analysed data from 133 patients with hearing aids, using the Abbreviated Profile of Hearing Aid Benefit (APHAB) and the Satisfaction with Amplification in Daily Life (SADL) questionnaires. Patients were divided into two groups based on this median age and sex for comparative analysis. The Mann Whitney test was applied with references to numerical parameters (APHAB and SADL) to assess differences between male vs female subjects and adults vs elderly. The Spearman correlation test was applied to assess the possible correlation between APHAB and SADL. A *p-*value lower than 0.05 was considered statistically significant.

**Results:**

Results indicated no significant gender differences in most APHAB and SADL subscales, except for the SADL “personal image” subscale, which showed lower scores for women (*p* =  0.023). Age-based comparison revealed no significant differences in APHAB scores; the SADL scores showed significant differences in the “positive effect” (*p* = 0.013), “negative features” (*p* = 0.005), and overall scores (*p* = 0.039), with lower satisfaction in older patients. Correlation analysis identified a positive relationship between the SADL “negative features” and APHAB “aversiveness” subscales (*p* = 0.042, correlation coefficient ρ = 0.176).

**Conclusions:**

The study highlights the importance of addressing subjective perceptions and aesthetic concerns in hearing aid adoption, emphasizing the need for personalized rehabilitation strategies to improve patient outcomes and satisfaction.

**Level of evidence:**

Level 3.

## Highlights

• Hearing loss impacts communication and mental health across ages.
• Stigma and aesthetics limit hearing aid use, notably among youth.
• Untreated hearing loss causes isolation and depression.
• Smaller, discreet hearing aids enhance user satisfaction.
• Research should diversify to explore adoption factors fully.

## Introduction

The hearing is one of the fundamental senses, crucial for daily life. From an epidemiological standpoint, all individuals can be affected by this condition: adverse events at birth, viral infections, exposure to noise sources, ototoxic medications, and aging are among the most common causes of hearing loss.^1^, ^2^

The World Health Organization (WHO) estimates that 1.5 billion people are affected by hearing loss.^1^

In 1991, the WHO proposed a classification system for categorizing hearing loss based on audiometric thresholds^3^, ^4^; this system has since undergone modifications by the Global Burden of Disease (GBD) Expert Group on Hearing Loss.^5^, ^6^

However, there also exists a European classification currently accepted and described by Uimonen et al.^4^

Approximately, 50% of individuals aged 60–69 years and 80% of those aged 85 and older have such severe hearing loss that it compromises communication ability.^7^ Hearing loss is the most common sensory disorder in the elderly population.^8^ This age-related decline in hearing is defined as Age-Related Hearing Loss (ARHL).^8^

Hearing loss has a negative psychosocial impact on the daily lives of individuals affected by it. Auditory deprivation results in language development delays in children and poses a risk factor for dementia and cognitive decline in adults.^5^

Today, there are various tools available that enable individuals with irreversible hearing loss to restore an adequate quality of hearing.^1^

One of the most common and effective rehabilitative approaches relies on the use of the “hearing technology”. This, utilizes specific devices designed to restore a good hearing quality, tailored to the type and degree of the hearing loss. These devices include hearing aids, implantable aids, and cochlear implants.^1^ The WHO estimates that currently, approximately 430 million people worldwide require hearing rehabilitation.^1^

Implantable aids are surgically implanted devices; among these, commonly employed ones are bone conduction implants used to treat conductive, mixed, or unilateral hearing loss and cochlear implants, utilized in the treatment of severe and profound hearing loss.^9^, ^10^

The most employed hearing devices today, however, are air-conduction hearing aids (conventional hearing aids). There exists a wide variety of devices which are available in different shapes and power levels, used in the treatment of mild to severe hearing loss. They are worn directly by the patient and amplify sounds from the external environment. Furthermore, the advent of digital technology has greatly enhanced listening capabilities, even in noisy environments.^11^

Based on these premises, an analysis was conducted on the data collected at our tertiary-level audiology centre from patients using conventional hearing aids. Since hearing loss and the use of hearing aids still represent significant stigmas in contemporary society, this study aimed to analyse the perceived level of audiologic benefit and satisfaction among conventional hearing aids adult users.

## Methods

### Participants

The study was conducted at the tertiary referral centre of the [blinded for review]. Data from 133 patients were collected between January 2022 and May 2024 and retrospectively analysed. Inclusion criteria included adult subjects over 18 years of age with conductive, sensorineural, or mixed hearing loss, fitted with conventional air conduction hearing aids. Only first-time hearing aid users with Behind-The-Ear (BTE) with Receiver-In-The-Ear (RITE) or Completely-In-the-Canal (CIC) devices were included.

The research adhered to the Declaration of Helsinki. This study represents a retrospective analysis of data obtained from our clinical practice and the protocol routinely applied by the centre. All patients provided their consent for the use of personal data. Due to the non-invasiveness of the tests, the use of a standard hearing aids application protocol, and the retrospective nature of the analysis, no ethical committee approval was required for the study’s execution.

### Subjective scales

To assess the psychological experience of these patients, two questionnaires validated in the scientific literature were used. Two aspects were evaluated: the auditory benefit provided by the hearing aid and the overall satisfaction experienced by the user during hearing aid use. To evaluate these aspects, the Abbreviated Profile of Hearing Aid Benefit (APHAB) and the Satisfaction with Amplification in Daily Life (SADL) questionnaires, designed and validated by Cox and Alexander (1995, 1999 respectively), were employed.^12^, ^13^

The APHAB questionnaire consists of 24 questions divided into four subscales: ease of communication, background noise, reverberation, and aversiveness.^12^ Each subscale includes six questions. The patient gives two scores from “A” (always) to “F” (never) for the same item (aided and unaided condition). The benefit is calculated by subtracting the aided score from the unaided score.^12^

For the SADL questionnaire, the patient gives a score from 1 (not at all) to 7 (tremendously) in response to 15 items.^13^ The items are divided into four groups to obtain four scores for positive effect, service and cost, negative features, and personal image subscales.^13^

### Procedure

The standard hearing aid fitting procedure followed in our centre was conducted. This involves performing an otomicroscopic examination. A standard pure-tone and speech audiometric examination is conducted, and a hearing aid is prescribed. The choice of hearing aid is made in agreement with the patient, considering their personal needs, aesthetics, costs, usage, etc.

During the initial fitting, in-situ audiometry with the hearing aid and feedback tests are then performed. The patient is seen weekly to ensure the achievement of the desired amplification gain within no less than four weeks.

Starting from the fifth week after the initial fitting, Real Ear Measurements (REMs) are performed.

At three months post-REMs, the patient undergoes to pure-tone and speech audiometry in a free field with the hearing aids worn and functioning, followed by the Italian matrix sentence test, also in a free field and with the hearing aids. This latter test is the most reliable tool for assessing the auditory outcomes of hearing aid users. The patient must repeat 20 sentences, each composed of five words, presented alongside background noise (fixed at 65 Db SPL) to simulate a competitive everyday listening condition. The Speech Reception Threshold (SRT) is obtained; it is the dB SNR level at which the patient can hear 50% of the presented words.

Also, at the three-month mark, patients complete the APHAB and SADL questionnaires. The decision to conduct these tests at third month is based on the need to provide the patient with an adaptation period to the new listening environment.

Since the objective of our study is to evaluate psychological aspects, we decided to analyse only these aspects by examining the questionnaire scores provided by the patients. For more details on the fitting process followed in our centre, see Portelli et al. 2024.^11^

### Statistical analysis

The numerical data were expressed as median and Interquartile range (Q1‒Q3), the categorical variables as absolute frequencies and percentage.

The non-parametric approach was used since variables were not normally distributed, such as verified by Kolmogorov Smirnov test.

In order to assess the existence of significant differences between male and female subjects and, in addition, between adults and elderly, the Mann Whitney test was applied with references to numerical parameters (APHAB subscales and total score, SADL subscales and total score). Some boxplots were realized to better visualize the data.

The Spearman correlation test was applied to assess the possible correlation between APHAB and SADL dimensions.

Statistical analyses were performed using SPSS 27.0 for Window package.

A *p-*value lower than 0.05 was considered to be statistically significant and was reported in bold in the text and in the table.

## Results

The sample consists of 133 patients (44.4% female and 55.6% male) (Table 1). Two groups were created, with patients equally distributed based on the median age of 69 years. Mean, standard deviation, and quartiles describe the numerical variables of age, the subscales, and the total score of the APHAB and SADL questionnaires (Table 2).

**Table 1:** Study population frequencies based on gender and age.

Sex	Frequency	Percentage
Females	59	44.4%
Males	74	55.6%
Total	133	100%
**Age (years)**		
≤ 69	67	50.4%
> 69	66	49.6%
**Total**	133	100%

**Table 2:** Descriptive statistics of numerical variables (age, APHAB and SADL subscales and total score).

		Percentiles		
	Mean ± SD	25	50	75
Age (years)	66.92 ± 14.82	62.00	69.00	76.00
APHAB ease of communication	42.23 ± 21.68	27.00	39.80	56.50
APHAB background noise	37.62 ± 18.58	25.15	35.80	49.00
APHAB reverberation	39.75 ± 19.37	27.00	35.50	51.25
APHAB aversiveness	−4.43 ± 16.49	−9.60	0.00	3.25
APHAB total score	39.87 ± 17.40	29.10	37.80	52.05
SADL positive effect	5.21 ± 1.17	4.70	5.50	6.00
SADL service and cost	4.96 ± 1.28	4.30	5.00	6.00
SADL negative features	4.63 ± 1.21	3.70	4.70	5.70
SADL personal image	5.63 ± 1.25	5.00	6.00	6.70
SADL Total score	5.13 ± 0.77	4.90	5.30	5.60

SD, Standard Deviation; APHAB, Abbreviated Profile of Hearing Aid Benefit; SADL, Satisfaction with Amplification in Daily Life.

**Fig. 1:**
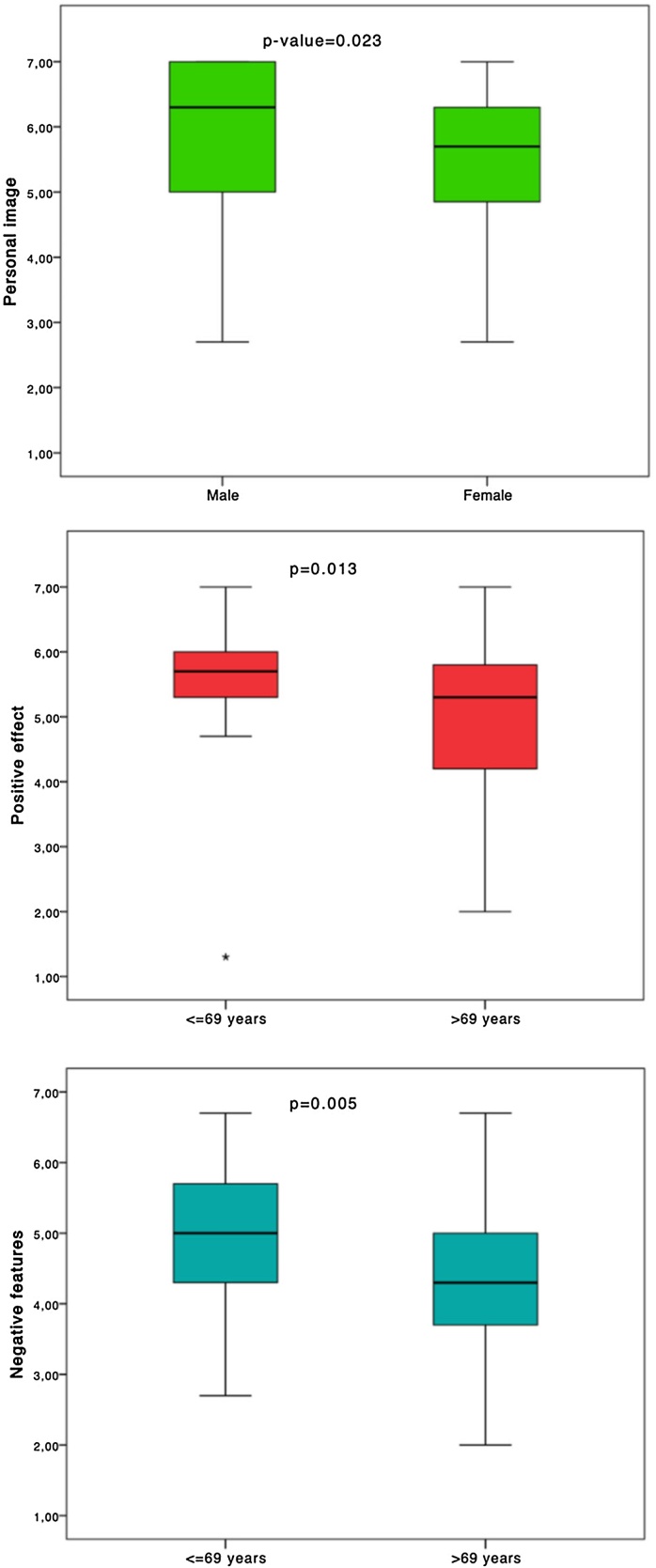
Boxplot for the distribution of scores on “Personal Image”, “Positive effect” and “Negative features” subscales of the SADL questionnaire, categorized by gender, and by age classes (≤69 vs. >69-years subjects).

Comparison of the total and subscale scores of the APHAB and SADL questionnaires between males and females showed no statistically significant differences, except for the “personal image” score of the SADL (*p* = 0.023), indicating that personal image has a greater impact on women than on men (lower scores in the personal image items for women) (Table 3, Fig. 1).

**Table 3:** Comparison of APHAB and SADL subscales and total score between males and females’ subjects.

	Male	Female	
	Mean ± SD	Mean ± SD	*p-*value
APHAB ease of communication	40.75 ± 22.57	44.08 ± 20.56	0.411
APHAB background noise	36.58 ± 18.27	38.94 ± 19.03	0.535
APHAB reverberation	38.23 ± 18.39	41.65 ± 20.54	0.487
APHAB aversiveness	−5.24 ± 17.57	−3.40 ± 15.10	0.796
APHAB total score	38.52 ± 17.22	41.56 ± 17.62	0.494
SADL positive effect	5.04 ± 1.27	5.44 ± 1.00	0.077
SADL service and cost	4.93 ± 1.34	4.99 ± 1.20	0.931
SADL negative features	4.53 ± 1.29	4.75 ± 1.11	0.277
SADL personal image	5.79 ± 1.29	5.43 ± 1.18	0.023*
SADL total score	5.06 ± 0.87	5.20 ± 0.62	0.732

SD, Standard Deviation; APHAB, Abbreviated Profile of Hearing Aid Benefit; SADL, Satisfaction with Amplification in Daily Life.

The same comparative analysis was performed by dividing the sample into two groups based on age: greater than 69 years and less than or equal to 69 years. No statistically significant differences were found for all subscales of the APHAB questionnaire and the total score. In contrast, the SADL questionnaire revealed differences in the “positive effect” subscale (*p* = 0.013), “negative features” subscale (*p* = 0.005), and the overall score (*p* = 0.039) (Table 4). Boxplot graphs illustrate these differences (Fig. 1).

**Table 4:** Comparison of APHAB and SADL subscales and total score between younger (≤69-years) and older (>69-years) subjects.

	≤69 years	>69 years	
	Mean ± SD	Mean ± SD	*p*-value
APHAB ease of communication	43.43 ± 23.38	41.01 ± 19.92	0.871
APHAB background noise	38.63 ± 19.68	36.60 ± 17.48	0.808
APHAB reverberation	41.56 ± 21.57	37.90 ± 16.82	0.420
APHAB aversiveness	−5.01 ± 18.97	−3.83 ± 13.63	0.948
APHAB total score	41.21 ± 19.30	38.50 ± 15.25	0.633
SADL positive effect	5.43 ± 1.13	4.99 ± 1.18	0.013*
SADL service and cost	4.96 ± 1.40	4.96 ± 1.15	0.641
SADL negative features	4.89 ± 1.16	4.37 ± 1.22	0.005*
SADL personal image	5.43 ± 1.31	5.83 ± 1.16	0.065
SADL total score	5.23 ± 0.77	5.02 ± 0.77	0.039*

SD, Standard Deviation; APHAB, Abbreviated Profile of Hearing Aid Benefit; SADL, Satisfaction with Amplification in Daily Life.

**Fig. 2:**
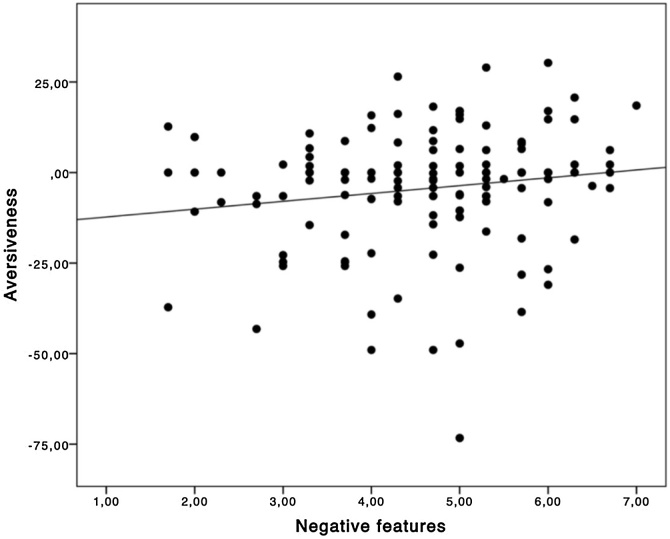
Positive correlation between the “Aversiveness” subscale of the APHAB questionnaire and the “Negative Features” subscale of the SADL questionnaire (correlation coefficient ρ = 0.176, *p*-value = 0.042).

Additionally, non-parametric correlations were conducted, highlighting a positive joint variation between the “negative features” subscale of the SADL and the “aversiveness” subscale of the APHAB (*p* = 0.042, correlation coefficient ρ = 0.176) (Fig. 2). A negative trend towards significance was observed when correlating the "aversiveness" score of the APHAB with the “personal image” score of the SADL (*p* = 0.066, correlation coefficient −0.160). Several scatter plots were used to illustrate the correlations between these variables.

## Discussion

Hearing loss is a prevalent condition affecting a significant portion of the world population. Hearing loss represents a global public health issue affecting both children and adults.^1^ The International Classification of Functioning, Disability and Health (ICF) defines deaf people across three dimensions: impairment, activity limitation, and participation restriction.^14^

The extent to which hearing loss impacts an individual's life depends on the adequacy of the rehabilitative intervention^15^; additionally, the presence of other comorbidities such as visual impairment, autism, or other disorders can influence functional recovery.^1^, ^15^

A patient with hearing loss must face various obstacles in daily life if the condition is not remedied. The primary issue is the challenge of maintaining adequate communication skills. This, of course, includes the ability to hear what is being communicated, which can range from struggling to hear verbal messages in noisy environments to being unable to hear even loud alarm noises.^16^

In children, inadequately corrected hearing loss leads to delays or even an inability to develop language skills.^17^

For adults and elderly individuals, as the subjects examined in our study, hearing loss contributes to social isolation, loneliness, anxiety, self-rejection, frustration, and anger.^18^, ^19^, ^20^

Given the above, auditory rehabilitation is crucial. It has been demonstrated that auditory rehabilitation leads to an overall improvement in quality of life and cognitive benefits.^21^ Conventional hearing aids are the most frequently used auditory rehabilitation devices; their choice depends on the type and degree of hearing loss. Additionally, considerations regarding the individual's communication needs, lifestyle, activities, psychological and emotional state, cognitive abilities, and financial resources must be made, as the selection of the hearing device necessarily takes these factors into account.^21^

However, despite the benefits that hearing aids provide, their usage remains low.^21^, ^22^ In recent years, progress has been made both in terms of the technologies used and the design of these devices. It is estimated that in industrialized countries, only 20%–30% of individuals with hearing loss wear hearing aids;^22^ among adults over seventy with hearing loss, less than 30% have ever used a hearing aid; for those between 20 and 69, less than 16% have used one.^23^

Various factors influence the adoption of hearing aids.^22^, ^23^, ^24^ Positive factors include the perception of disability, the severity of hearing loss, activity limitation, and socioeconomic status.^22^ Conversely, predisposition and stigma are negative factors affecting their use. Reluctance to believe in the benefits that hearing aids can provide is one of the main reasons for not using them.^22^

Goffman (1963) defines stigma as “an attribute that is deeply discrediting” that reduces someone “from a whole and usual person to a tainted, discounted one”.^22^, ^23^, ^24^, ^25^ The aesthetic aspect significantly influences the adoption rate of hearing aids, as they make deaf people feel “disabled” or “old”.^22^, ^26^, ^27^, ^28^

This stigma is primarily linked to the aesthetic aspects of hearing aids, which are often viewed as bulky and noticeable.

However, modern hearing aids are becoming increasingly small and discreet. For example, hearing aids such as Invisible-In-Canal (IIC) or extended wear hearing aids are placed near the tympanic membrane and are virtually invisible.^29^ It is important to note that each device or prescription rule has its limitations.^11^ Such innovations are gradually changing public perceptions and encouraging more people to seek help for their hearing loss.

In our study, we aimed to evaluate the subjective aspect of the benefit and satisfaction provided by the use of hearing aids. The assessment of subjective benefit was conducted using the APHAB questionnaire. In our case, all patients, prior to the administration of the questionnaire, underwent Real Ear Measurements (REM) and the matrix sentence test. REMs allow for direct recording within the patient's external ear canal while wearing and operating the hearing aid to determine if the amplification provided is appropriate for the patient's audiometric threshold, ensuring it reaches the target curve.^11^ The matrix sentence test assesses the patient's listening abilities while wearing the hearing aid.^30^ These tests are now routinely used in clinical practice and during the hearing aid fitting process.

However, based on our experience, it often happens that despite these tests yielding positive results, the patient does not perceive a real benefit (even if it is present). They may often feel dissatisfied. We believe that prejudices and subjective perceptions that individuals with hearing loss have, can negatively impact the degree of hearing aid adoption.

We compared the questionnaire scores between male and female subjects observing the absence of statistically significant differences for all scores of the APHAB and SADL questionnaires, except for the “personal image” subscale of the SADL, which was statistically different. It is frequently observed during audiological counselling that women have more reservations about adopting hearing aids due to the perceived aesthetic defect these devices might cause. Women tend to choose hearing aids that are less noticeable, such as CIC, Invisible-In-the-Canal (IIC), or extended wear devices. Therefore, the result obtained can be considered reliable and expected.

The same comparative analysis was conducted by dividing the patients into two groups based on the median age of the sample (69-years). Regarding the benefit, measured by the APHAB questionnaire scores, no significant difference was found between subjects aged 69-years or younger and those older than 69-years. However, for the SADL questionnaire scores, differences were noted in the positive effect, negative features, and consequently the overall score, with statistically lower scores in these subscales for older people. Cox and Alexander (1999) include two dimensional aspects in the positive effect: acoustic and psychological.^13^ These items refer to a reduction in communicative hearing disability, improvement in sound localization and naturalness, also encompassing the psychological satisfaction level.^13^ Regarding negative features, the same authors refer to aspects that make hearing aid use less satisfying (e.g., feedback, difficulty hearing sounds, etc.).^13^ In this regard, it is important to remember that the aging process negatively affects overall listening performance, as these abilities are significantly dependent on the mental status of the individual.^31^ Elderly individuals, due to their comorbidities (not only neurological but also other types), may have greater difficulty managing their hearing aids daily or may need assistance with this. Consequently, older subjects might show a lower degree of satisfaction precisely because of these factors. However, it should be noted that this result was not confirmed by the APHAB questionnaire, which is more precise in evaluating audiological benefits.

From the correlation study, a positive interdependence was observed between the APHAB subscale “aversiveness” and the SADL “negative features”. This result is predictable and aligns with our expectations. Another noteworthy finding is the trend towards a correlation between the “aversiveness” subscale of the APHAB and the “personal image” subscale of the SADL, which approaches but does not reach statistical significance. As the “personal image” score increases, the “aversiveness of sounds” score decreases. This subscale refers to negative reactions to environmental sounds. At first glance, one might think these factors are not related; however, we hypothesize that an underlying psychological aspect could explain this result. Our hypothesis suggests that biases and the degree of aesthetic satisfaction play a role. A person who accepts wearing a hearing aid and is satisfied with its aesthetic appearance might better tolerate ambient noises and find them less annoying. This result could also apply to other APHAB subscales (e.g., reverberation and background noise), although we must remember that modern hearing aids, with their advanced digital sound processing technology, minimize these effects.^32^

Our study is based on a retrospective analysis of data collected at our centre. This could be a limitation of the study, as it only examines a small geographic area. The degree of hearing aid adoption, benefit, and overall satisfaction among users could be influenced by various factors, particularly geographic ones. Conducting a multicentre study including more subjects, could provide a more realistic estimate. Another limitation is that only patients using conventional hearing aids were considered; patients with bone conduction devices were not analysed. Furthermore, children and younger subjects were not included, and their subjective assessments could vary due to different needs and requirements. It is through these data and the study of subjective aspects that factors influencing the adoption of hearing aids can be identified.

Furthermore, this study is based on data collected from modern hearing aids, which feature digital sound processing that enhances listening quality even in noisy environments. The increasingly smaller size of modern hearing aids makes these devices more acceptable. Therefore, we believe it is important to investigate the factors that sustain the current and persistent stigma regarding the non-adoption of hearing aids.

## Conclusions

The effects of hearing loss on an individual's life can be profound, influenced by both the adequacy of rehabilitative interventions and the presence of comorbidities.

Our study revealed significant insights into the demographics and psychological aspects influencing hearing aid adoption.

Gender differences were noted, with women expressing greater concerns about the aesthetic aspects of hearing aids. Additionally, age-related differences indicated that older individuals reported lower satisfaction levels with their hearing aids.

Overall, the primary goal remains the fitting and adoption of hearing aids to ensure effective auditory rehabilitation.

## Ethical approval

The research adhered to the Declaration of Helsinki. This study represents a retrospective analysis of data obtained from our clinical practice and the protocol routinely applied by the centre. All patients provided their consent for the use of personal data. Due to the non-invasiveness of the tests, the use of a standard hearing aids application protocol, and the retrospective nature of the analysis, no ethical committee approval was required for the study’s execution.

## Declaration of Generative AI and AI-assisted technologies in the writing process

During the preparation of this work the author(s) used ChatGPT only for English translation purpose. After using this tool/service, the author(s) reviewed and edited the content as needed and take(s) full responsibility for the content of the published article.

## Funding

This research did not receive any specific grant from funding agencies in the public, commercial, or not-for-profit sectors.

## Declaration of competing interest

The authors declare no conflicts of interest.
